# Alzheimer’s pathology in South African people ageing with HIV: the Ingqondo study

**DOI:** 10.1002/alz70862_109840

**Published:** 2025-12-23

**Authors:** Ziphozihle Ntwatwa

**Affiliations:** ^1^ University of Cape Town, Cape Town, Rondebosch South Africa

## Abstract

**Background:**

Dementia prevalence is increasing globally, with low‐ and middle‐income countries expected to bear the greatest burden due to increased longevity and differing comorbidities. HIV can cause brain injury and is associated with chronic neuroinflammation, even when treated. South Africa has the largest population of people living with HIV in the world; a cohort that is ageing due to antiretroviral therapy success. The risk of dementia in people ageing with HIV is not clear. There is evidence supporting neuroinflammation underlying Alzheimer’s disease (AD) pathogenesis; how HIV interacts with AD pathology is also unknown.

**Method:**

Two studies in our group aimed to investigate the role of HIV in dementia in older persons: a previous community‐based dementia screening study survey and the new Ingqondo study, a deep phenotyping study to explore mechanisms. Our community survey screened 1150 participants (mean age 71 years) in rural South Africa with the brief Community Screening Instrument for Dementia (CSID), 4.8% of whom were living with HIV. The Ingqondo study uses the full CSID and informant report (10/66 algorithm) in 250 participants ≥70 years old in a low‐income peri‐urban area of Cape Town, 50% of whom are living with HIV. Ingqondo participants undergo neuroimaging (including diffusion weighted MR spectroscopy to measure neuroinflammation) and sampling of blood and cerebrospinal fluid for viral, AD and inflammatory biomarkers.

**Results:**

127/1150 (11.0%) in the rural community survey screened positive for dementia (18.18% of persons with HIV vs. 10.68% without HIV). In adjusted analyses, HIV+ status, older age and depression were associated with higher dementia risk (ps<.04). The Ingqondo study has to date recruited 33 participants without HIV (median age 76 years). Preliminary analysis indicates 7 (21.2%) participants have screened positive for dementia, with no differences in age, gender or education level between those with and without dementia.

**Conclusions:**

HIV is associated with all‐cause dementia in older persons. The study of people ageing with HIV (Ingqondo) offers a unique opportunity to explore how chronic infection and neuroinflammation may contribute to AD pathology. This could inform AD interventions, and help to understand how to maintain brain health for the increasing numbers of people ageing with HIV globally.

